# The impact of nimodipine combined with Ginkgo biloba extract on cognitive function and ADL scores in patients with Parkinson’s disease: A retrospective study

**DOI:** 10.1097/MD.0000000000038720

**Published:** 2024-07-19

**Authors:** Lianlian Zhang, Hua Sun, Zaigang Han

**Affiliations:** a Department of Pharmacy, Affiliated Hospital of Beihua University, Jilin, China; b Department of Endocrinology, Affiliated Hospital of Beihua University, Jilin, China.

**Keywords:** nimodipine, Parkinson’s disease, propensity score matching, retrospective study

## Abstract

This study aims to explore the value of nimodipine combined with Ginkgo biloba extract in improving cognitive function and daily living abilities in patients with Parkinson’s disease. Clinical data from 551 patients with Parkinson’s disease admitted to the Neurology Department of the Affiliated Hospital of Beihua University from January 2022 to December 2022 were retrospectively collected. Cognitive function and daily living abilities were assessed in patients before treatment, and a reevaluation was conducted after 12 weeks of medication. Patients treated solely with nimodipine were categorized into the monotherapy group, while patients treated with nimodipine combined with Ginkgo biloba extract were included in the combination group. After 1:1 propensity score matching, a total of 83 pairs of patients were matched, and differences in relevant indicators between the 2 groups were compared. The total effective rate of treatment in the combination group was 90.36%, which was higher than the control group at 72.29% (*P* < .05). However, after treatment, the observation group showed higher Mini-Mental State Examination and activities of daily living scores compared to the control group (*P *< .05). The combined treatment of nimodipine and Ginkgo biloba extract in patients with Parkinson’s disease has a significant effect and can effectively improve cognitive function and enhance daily living abilities.

## 1. Introduction

Parkinson’s disease is a progressive neurodegenerative disorder characterized by tremors and bradykinesia, and it is a common neurological condition.^[1]^ Gender and aging are independent risk factors, with the disease being less common in patients under the age of 40, but the incidence increases with advancing age.^[2]^ Several studies have shown that Parkinson’s disease typically onsets in men approximately 2 years earlier than in women, and the prevalence is about twice as high in men compared to women.^[3,4]^ From 1990 to 2015, the global population of Parkinson’s disease patients increased by 118%, leading to significant societal and healthcare burdens.^[5,6]^ Unlike many other neurodegenerative diseases, idiopathic Parkinson’s disease has effective treatments for symptom relief, with medications that can improve daily functioning.

Nimodipine is an L-type calcium channel antagonist primarily used for cardiovascular-related conditions, including angina, arrhythmias, and hypertension.^[7]^ Additionally, nimodipine has shown benefits in various central nervous system disorders, including stroke, brain injury, cerebral ischemia, epilepsy, dementia, and age-related degenerative diseases,^[8,9]^ and has been found effective in the treatment of dementia in the elderly.^[10]^ Nimodipine has also found wide application in the clinical treatment of Parkinson’s disease.

Ginkgo biloba leaf extract is derived from the dried leaves of the Ginkgo biloba tree and is used clinically for the treatment of memory impairments and dementia, including Alzheimer’s disease (AD).^[11,12]^ Many clinical studies have indicated improvements in cognitive function in elderly individuals and AD patients following Ginkgo biloba treatment.^[13,14]^

Propensity score analysis is a widely used method in the fields of statistics and econometrics to address confounding in observational studies.^[15]^ The propensity score is the conditional probability of assignment to a particular treatment group given a set of observed covariates. It allows researchers to balance covariates between treatment and control groups, making it easier to estimate causal effects when randomization is not possible. The process typically involves estimating the propensity scores for each individual, which are then used to match or weight the data to create groups that are comparable in terms of covariate distribution. Subsequently, outcome analyses are performed on these balanced groups to estimate the treatment effect.

Nimodipine and Ginkgo biloba leaf extract have been widely applied in the treatment of Parkinson’s disease. However, there is currently no research demonstrating that the combined use of these 2 drugs is more effective than using nimodipine alone. This study is based on retrospective data using a propensity scoring method to explore the value of combining nimodipine with Ginkgo biloba leaf extract in improving cognitive function and daily living abilities in Parkinson’s disease patients.

## 2. Materials and methods

### 2.1. Data source

The study was carried out in conformity with the ethical guidelines of the Declaration of Helsinki, and it received approval from the Ethics Committee of the Affiliated Hospital of Beihua University (ID#20211008). Retrospective Collection of Data from the Neurology Department at the Affiliated Hospital of Beihua University for Parkinson’s Disease Patients from January 2022 to December 2022. Based on the inclusion and exclusion criteria, a total of 551 eligible patients were selected. Patients treated only with nimodipine were included in the Monotherapy Group, while patients treated with a combination of nimodipine and Ginkgo biloba extract were included in the Combination Group. Clinical data, including gender, age, smoking status, pre-treatment Unified Parkinson’s Disease Rating Scale (UPDRS) score, Mini-Mental State Examination (MMSE) score, activities of daily living (ADL) score, post-treatment UPDRS score, MMSE score, and ADL score, were collected.

### 2.2. Inclusion and exclusion criteria

Inclusion criteria: ① confirmed diagnosis of Parkinson’s disease based on medical history, clinical symptoms, and signs; ② MMSE score <24 points; ③ stable cardiopulmonary function.

Exclusion criteria: ① severe organ diseases, such as heart, brain, and lung conditions; ② patients with limb paralysis; ③ patients with severe visual or hearing impairments.

### 2.3. Methods

All patients included in this study received routine symptomatic treatment, including levodopa and pramipexole. In addition to the above treatment, the Combination Group received nimodipine in combination with Ginkgo biloba extract. The Control Group was treated with nimodipine alone. The daily dose ranged from 30 to 120 mg, taken in 3 divided doses, continuously for 3 months.

### 2.4. Assessment of therapeutic efficacy

The improvement in the UPDRS scores before and after treatment was used to determine therapeutic efficacy. The improvement rate was calculated as follows: improvement rate = (pre-treatment score ‐ post-treatment score)/pre-treatment score × 100%. An improvement rate of 100% indicated a cure, 50% to 100% was considered highly effective, 20% to 50% was effective, and <20% was considered ineffective.^[16]^

### 2.5. Observation metrics

The MMSE and ADL^[17]^ scale were used to evaluate cognitive function and the ability to perform daily activities before and after treatment for both groups, with higher scores indicating better performance.

### 2.6. Statistical methods

Continuous variables were described as mean ± standard deviation or median and interquartile range according to the distribution of data. Categorical variables were described as frequency and percentage. Qualitative variables were compared with Pearson’s Chi-Squared test or Fisher’s exact test as appropriate. Continuous variables were compared with Two-Independent-Sample Tests or Mann–Whitney *U* Test. R 4.2 statistical software was used for propensity score matching based on gender, age, UPDRS score, MMSE score, and ADL score, with a matching tolerance of 0.02. The statistical significance in all analyses was set at a *P*-value ≤ .05.

## 3. Results

### 3.1. Demographic characteristics

In this study, a total of 551patients were included, with 265 patients in the Combination group and 286 patients in the Single Drug group. Prior to propensity score matching, the Combination group had a higher mean age of 74.39 compared to 70.07 in the Single Drug group (*P* < .001). The proportion of male patients in the Combination group was 82.64%, which was higher than the 61.89% in the Single Drug group (*P* < .001). The Combination group had a higher UPDRS score of 54 before treatment, compared to 48 in the Single Drug group (*P* < .001). The MMSE score for the Combination group was 15, which was lower than the 17 in the Single Drug group (*P* < .001). The ADL score for the Combination group was 73, lower than the 76 in the Single Drug group (*P* < .001). After propensity score matching, the baseline characteristics of the 2 groups were balanced. There were no significant differences between the 2 groups in terms of age, gender, UPDRS score, MMSE score, and ADL score (Table 1).

**Table 1:** Characteristics of combination group and single drug groups before and after PSM.

Characteristics	Before PSM		*P*	After PSM		*P*
	Combination	Single drug		Combination	Single drug	
Age	74.39 ± 10.87	70.07 ± 11.57	<.001	72.23 ± 11.01	72.82 ± 10.73	.727
Gender (male）	82.64%	61.89%	<.001	79.49%	74.70%	1
Smoke	63.77%	36.71%	<.001	45.78%	53.01%	.438
Pre-treatment UPDRS	54 (51, 57)	48 (45, 52)	<.001	52 (49, 55)	52 (49, 54)	.58
Pre-treatment MMSE	15 (13, 16)	17 (16, 19)	<.001	16 (14, 18)	16 (14, 17)	.555
Pre-treatment ADL	73 (69, 78)	76 (73, 79)	<.001	74.66 ± 4.15	74.96 ± 6.83	.732

ADL = activities of daily living; MMSE = Mini-Mental State Examination; PSM = propensity score matching; UPDRS = Unified Parkinson’s Disease Rating Scale.

**Figure 1.**
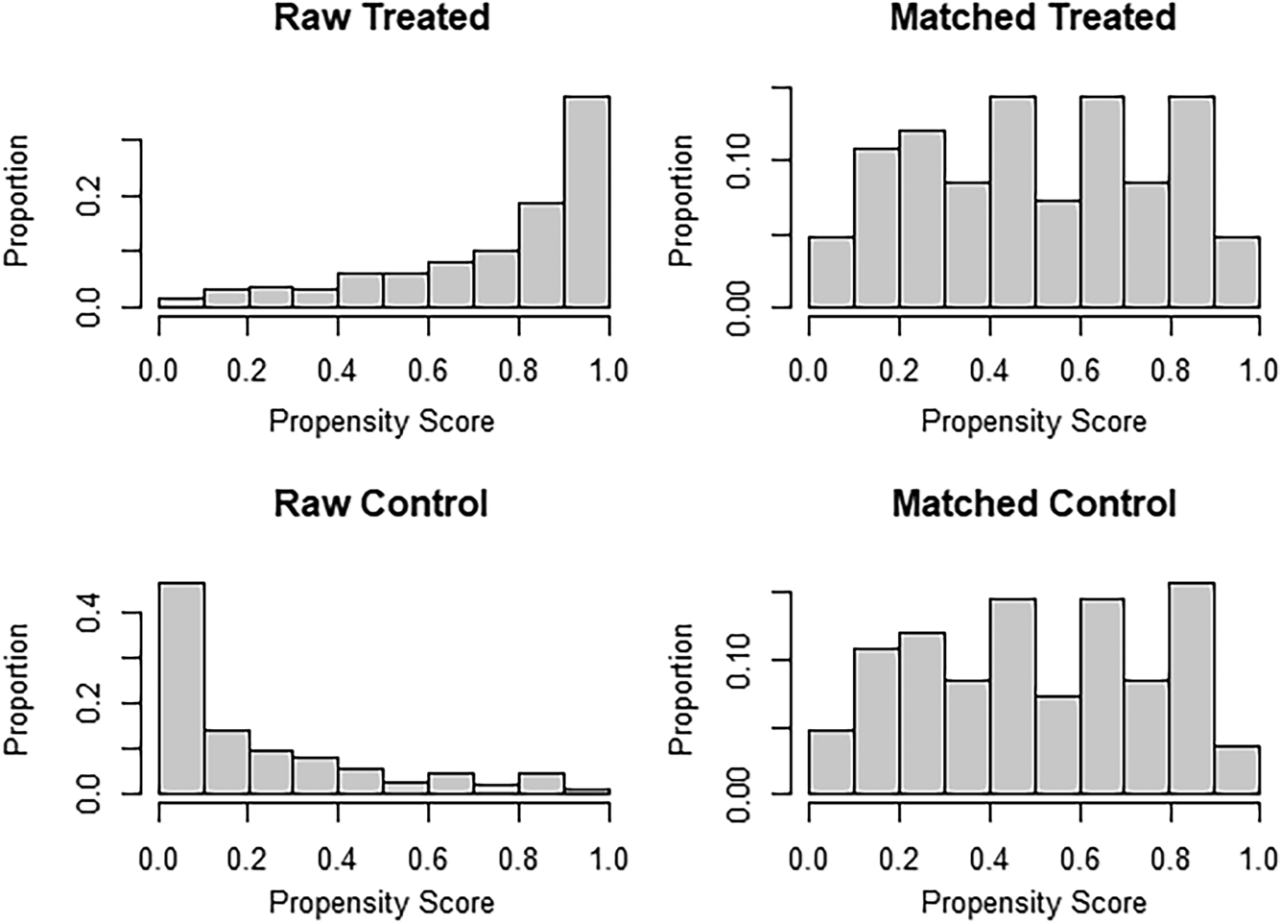
Matching anterior–posterior propensity score bars.

The propensity score histogram visually represents the distribution of propensity scores in our dataset (Fig. 1). Propensity scores, calculated based on observed covariates, are essential for ensuring comparability between treatment and control groups in observational studies. In this histogram, the *x*-axis represents the propensity scores, ranging from the lowest to the highest values, while the *y*-axis indicates the frequency or count of individuals within each propensity score interval. Upon examination of the histogram, we observe that the propensity scores are well-distributed across the range of values, indicating that the treatment and control groups are balanced in terms of their propensity to receive the treatment. A balanced distribution of propensity scores is crucial as it suggests that potential confounding variables have been adequately controlled for, enhancing the validity of our causal inference.

### 3.2. Clinical efficacy

The total treatment effectiveness in the observation group was 90.36%, which was higher than the control group’s 72.29% (χ^2^ = 8.925, *P* = .003). See Table 2 for details.

**Table 2:** Comparison of clinical efficacy between the 2 groups.

Group	n	Cure	Remarkable	Effective	Ineffective	Total effective rate
Combination	83	10	57	8	8	90.36%
Single drug	83	5	40	15	23	72.29%

### 3.3. Functional assessment

**Figure 2.**
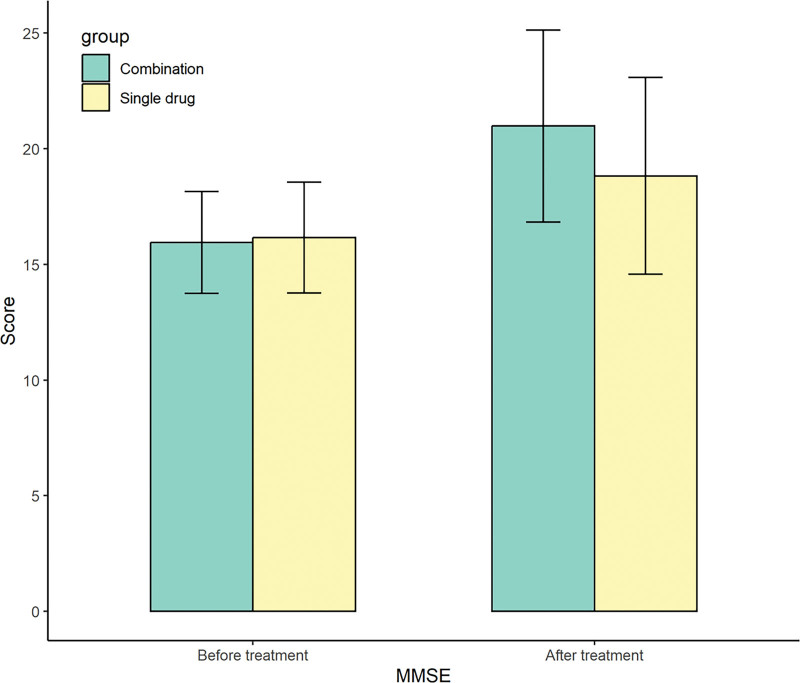
Changes of MMSE scores before and after treatment between the 2 groups. MMSE = Mini-Mental State Examination.

**Figure 3.**
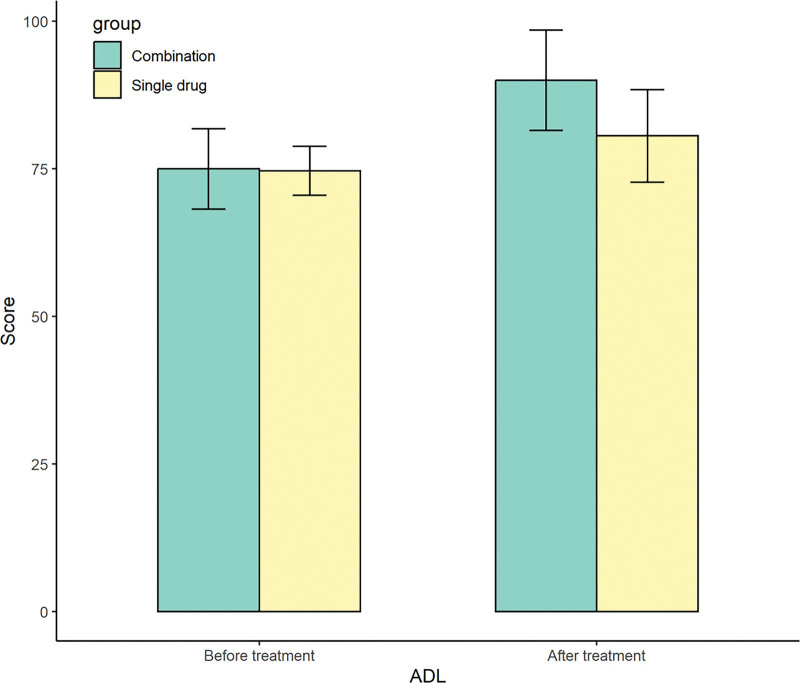
Changes of ADL scores before and after treatment between the 2 groups. ADL = activities of daily living.

Before treatment, there were no statistically significant differences in MMSE scores and ADL scores between the 2 groups (*P* > .05). After treatment, the MMSE scores and ADL scores in the observation group were higher than those in the control group (*P* < .05). See Table 3 for details. The pictures clearly show the difference between the 2 groups before and after treatment (Figs. 2 and 3).

**Table 3:** Comparison of MMSE and ADL scores in 2 groups before and after treatment.

Group	n	MMSE scores		ADL scores	
		Pre-treatment	Pro-treatment	Pre-treatment	Pro-treatment
Combination	83	16 (14, 18)	74.66 ± 4.15	18.82 ± 4.25	80.55 ± 7.86
Single drug	83	16 (14, 17)	74.96 ± 6.83	20.98 ± 4.15	90 ± 8.5
*P*		.555	.732	.001	<.001

ADL = activities of daily living; MMSE = Mini-Mental State Examination.

## 4. Discussion

In this study, based on retrospective data, we employed propensity score matching to compare baseline data between the group treated with nimodipine in combination with Ginkgo biloba leaf extract and the group treated with nimodipine alone. The application of propensity score matching in our retrospective medical study has yielded several significant advantages that have enhanced the robustness and credibility of our findings. Propensity score matching has effectively balanced the distribution of observed covariates between the treatment and control groups. This balance mitigates the impact of potential confounders, making our study results more trustworthy and allowing for a more accurate estimation of the treatment effect. We assessed and compared the clinical efficacy, cognitive function, and daily living abilities of the 2 groups. The findings indicate that the combination group exhibited superior effects compared to the monotherapy group. This suggests that clinicians treating Parkinson’s disease may consider the combination of nimodipine and Ginkgo biloba leaf extract as a potential therapeutic option.

Parkinson’s disease (PD) is a common and multifactorial clinical condition. Traditionally, clinical research has primarily focused on motor symptoms such as tremors and movement disorders. However, recent advances in neurobiology and functional neuroimaging have led to a deeper understanding of cognitive impairments in PD patients. The main mechanisms underlying PD pathogenesis include mitochondrial dysfunction, apoptotic events, oxidative and nitrosative stress, genetic susceptibility, protein aggregation, and proteasomal dysfunction.^[18–21]^ In the advanced stages of PD, there is a heightened risk of developing dementia, which significantly impairs the patients’ daily lives and activities, leading to a marked reduction in their quality of life. Consequently, effective interventions to mitigate cognitive impairments in PD patients and improve cognitive function have become a focus and priority in clinical research.

Epidemiological evidence suggests that the use of dihydropyridine calcium channel blockers in the treatment of hypertension is associated with a reduced risk of developing PD.^[22,23]^ Dihydropyridines are considered suitable candidates for slowing down or inhibiting the pathogenic mechanisms of PD because they retain binding sites for L-type calcium channels in the PD brain.^[24]^ Nimodipine, a calcium ion antagonist, has already achieved consensus in clinical practice for its efficacy and feasibility in the treatment of vascular dementia. Recent research has indicated a close relationship between cognitive impairment in PD and reduced cerebral blood flow, which nimodipine addresses by dilating cerebral blood vessels, increasing cerebral blood flow, improving brain metabolism, protecting neuronal synapses, reducing oxidative stress, and inhibiting lipid peroxidation. As a result, nimodipine plays a crucial role in improving cognitive function in PD patients.

Ginkgo biloba leaf extract (GBE) is known to contain 6% terpene compounds, 24% flavonol glycosides, and 5% to 10% organic acids. Flavonoids and terpene compounds are recognized as the pharmacologically active constituents of Ginkgo biloba leaf extract.^[25]^ The water solubility of GBE is attributed to the presence of organic acids.^[26]^ Significant research by Y. Luo and colleagues has demonstrated the neuroprotective properties of this herbal extract in both cellular and animal models. Various preclinical studies have been conducted to assess the effects of GBE.^[27,28]^ GBE has found widespread use in the treatment and prevention of neurodegenerative conditions associated with aging, AD, PD, peripheral vascular diseases, and sensory disorders like tinnitus.

While this study suggests that the combination of nimodipine and Ginkgo biloba leaf extract is more effective in treating Parkinson’s patients compared to nimodipine alone, it is essential to note that this is a retrospective study, and the strength of evidence is not as robust as that from randomized controlled trials. Therefore, the next step should involve conducting clinical trials to validate these findings. Additionally, it is imperative to delve deeper into the mechanisms of action of nimodipine in combination with Ginkgo biloba leaf extract to better understand the principles behind their therapeutic effects in Parkinson’s disease. This deeper understanding can potentially lead to improved quality of life for Parkinson’s patients.

## Author contributions

**Conceptualization:** Lianlian Zhang, Hua Sun, Zaigang Han.

**Data curation:** Lianlian Zhang, Hua Sun, Zaigang Han.

**Formal analysis:** Lianlian Zhang, Hua Sun, Zaigang Han.

**Funding acquisition:** Lianlian Zhang, Hua Sun, Zaigang Han.

**Investigation:** Lianlian Zhang, Hua Sun, Zaigang Han.

**Methodology:** Lianlian Zhang, Hua Sun, Zaigang Han.

**Project administration:** Lianlian Zhang, Hua Sun, Zaigang Han.

**Resources:** Lianlian Zhang, Hua Sun, Zaigang Han.

**Software:** Lianlian Zhang, Hua Sun, Zaigang Han

**Supervision:** Lianlian Zhang, Hua Sun, Zaigang Han

**Validation:** Lianlian Zhang, Hua Sun, Zaigang Han

**Visualization:** Lianlian Zhang, Hua Sun, Zaigang Han

**Writing – original draft:** Lianlian Zhang, Hua Sun, Zaigang Han.

**Writing – review & editing:** Lianlian Zhang, Hua Sun, Zaigang Han.
